# Editorial: Population and action mechanism of immune cells in fish

**DOI:** 10.3389/fimmu.2024.1425155

**Published:** 2024-05-24

**Authors:** Zejun Zhou, Yu Huang, Ruijuan Hao, Chuangye Yang, Emmanuel Delwin Abarike, Jun Li

**Affiliations:** ^1^State Key Laboratory of Developmental Biology of Freshwater Fish, Engineering Research Center of Polyploid Fish Reproduction and Breeding of the State Education Ministry, College of Life Sciences, Hunan Normal University, Changsha, Hunan, China; ^2^Guangdong Provincial Key Laboratory of Aquatic Animal Disease Control and Healthy Culture, Guangdong Ocean University, Zhanjiang, Guangdong, China; ^3^Development and Research Center for Biological Marine Resources, Southern Marine Science and Engineering Guangdong Laboratory (Zhanjiang), Zhanjiang, China; ^4^Department of Fisheries and Aquatic Resources Management, University for Development Studies, Tamale, Ghana; ^5^School of Science and Medicine, Lake Superior State University, Sault Ste. Marie, MI, United States

**Keywords:** fish, immune cells, innate immunity, adaptive Immunity, pathogen infection

Fish occupy a foundational position in vertebrate phylogeny, making them valuable subjects for comparative immunological studies. Mammalian immune system comprises various cell types, including monocytes/macrophages, granulocytes, natural killer cells, B cells, and T cells, which collectively contribute to both innate and adaptive immunity. Fish also have these cell types, but their action mechanisms are different from those of mammals. In this Research Topic, five articles including one review article and four original research articles highlight the recent advances in immune cells of the innate and adaptive immune systems of fish.

It is assumed that the innate immune system originated approximately 600 million years ago (MYA); whereas key components of the adaptive immune system, such as T cell receptors (TCR), emerged around 450 MYA in the first jawed vertebrates (i.e., *Gnathostomata*). *Gnathostomes* are divided into *Chondrichthyes* (cartilaginous fish) and *Osteichthyes* (bony fish). Smith et al. summarized the main components of the innate and adaptive immunity in *Chondrichthyes* and *Osteichthyes*. The authors further discussed the functions of these major components including macrophages, neutrophils, B cells and T cells in fish, which will contribute to our understanding of the evolution of the immune system.

The mammalian complement system is composed of the classical pathway, the alternative pathway and the lectin pathway. Most mammalian complement components have homologs in teleost, however, more research is needed to determine how complement components affect the function of immune cells in fish. In this Research Topic, Mu et al. discovered a mannose-binding lectin associated protein MAp34, OnMAp34, in Nile tilapia. They found that OnMAp34 is involved in non-specific cellular immune defenses of monocytes/macrophages, including inflammation, migration, and phagocytosis, and it can also compete with MBL-associated serine proteases (MASPs) to inhibit the activation of the lectin pathway. These results illustrated a novel mechanism by which OnMAp34 acts as a dual regulator of the lectin complement pathway and the function of monocytes/macrophages in fish. Upon viral infection, the host’s immune system recognizes pathogen-associated molecular patterns (PAMPs) through immune cells such as neutrophils and macrophages. This recognition leads to the activation of these cells and the production of antiviral effector molecules, such as Viperin. Shanaka et al. demonstrated that Viperin inhibited the viral replication during the infection of hemorrhagic septicemia virus (VHSV) in zebrafish. Interestingly, they found that Viperin-deficient zebrafish harbored disturbed lipid metabolisms and had higher cholesterol levels during VHSV infection. This study suggested that zebrafish already have an antiviral immune mechanism similar to that of higher vertebrates.

A general understanding of adaptive immunity in bony fish is that due to the absence of bone marrow and germinal centers (GC), progenitor B cells and plasma cells reside in the head kidney, while other mature B cells and plasma cells can be seen in the posterior kidney and spleen. Although some mechanisms have been proposed, further research is required to fully elucidate the mechanisms regulating homing of B cells in fish. For example, Blimp1 (encoded by *prdm1*) and Hobit (encoded by *znf683*) are key transcriptional factors that control the development and differentiation of B cells in mammals, but their function in fish is still largely unclear. In this context, a complete phylogenetic analysis of the *prdm1* family in fish has been performed by Perdiguero et al., and they found that *prdm1* and *znf683* evolved from a common ancestral gene and underwent genome duplication events in rainbow trout. Transcriptional studies further revealed that these genes are regulated in response to viral infection and during B cell differentiation, providing insight into their potential role in the immune response of teleost. Moreover, secreted novel AID/APOBEC-like deaminases (SNADs) play a crucial role in antibody diversity and antiviral defense in mammals. Until now, it was largely unknown whether SNADs are involved in immunity in teleost. In this Research Topic, Majewska et al. found that SNADs have a widespread presence in fish, with expression starting post-hatching and being highest in immune-related organs. Their results showed that SNAD1 exhibited changed expression levels in response to infection or temperature fluctuation, suggesting that SNADs may play an important role in the immune response of fish, possibly by influencing B cell activity and other immune cell functions.

In summary, the insights presented in this Research Topic will broaden our understanding of fish innate and adaptive immunity. We hope that these insights can serve as a source of inspiration for understanding the evolution of immune systems in vertebrates.

## Author contributions

ZZ: Funding acquisition, Writing – original draft, Writing – review & editing. YH: Funding acquisition, Writing – review & editing. RH: Writing – review & editing. CY: Writing – review & editing. EA: Writing – review & editing. JL: Writing – review & editing.

